# Quantitative and qualitative consequences of reduced pollen loads in a mixed‐mating plant

**DOI:** 10.1002/ece3.5858

**Published:** 2019-12-02

**Authors:** Laura S. Hildesheim, Øystein H. Opedal, W. Scott Armbruster, Christophe Pélabon

**Affiliations:** ^1^ Department of Biology Centre for Biodiversity Dynamics Norwegian University of Science and Technology NTNU Trondheim Norway; ^2^ Department of Biological Sciences University of Bergen Bergen Norway; ^3^ School of Biological Sciences University of Portsmouth Portsmouth UK; ^4^ Institute of Arctic Biology University of Alaska Fairbanks AK USA

**Keywords:** *Dalechampia scandens*, genetic sampling, pollen competition, pollinator decline, seed mass, seed set

## Abstract

Greater pollination intensity can enhance maternal plant fitness by increasing seed set and seed quality as a result of more intense pollen competition or enhanced genetic sampling. We tested experimentally these effects by varying the pollen load from a single pollen donor on stigmas of female flowers of *Dalechampia scandens* (Euphorbiaceae) and measuring the effects on seed number and seed mass. Seed set increased rapidly with pollen number at low to moderate pollen loads, and a maximum set of three seeds occurred with a mean pollen load of 19 pollen grains. We did not detect a trade‐off between the number of seeds and seed mass within a fruit. Seed mass increased with increasing pollen load, supporting the hypothesis of enhanced seed quality via increased pollen‐competition intensity or genetic sampling. These results suggest that maternal fitness increases with larger pollen loads, even when the fertilization success is already high. Our results further highlight the importance of high rates of pollen arrival onto stigmas, as mediated by reliable pollinators. Comparing the pollen‐to‐seed response curve obtained in this experiment with those observed in natural populations suggests that pollen limitation may be more severe in natural populations than predicted from greenhouse studies. These results also indicate that declines in pollinator abundance may decrease plant fitness through lowered seed quality before an effect on seed set is detected.

## Graphical abstract

Greater pollination intensity can enhance maternal plant fitness by increasing seed set and seed quality as a result of more intense pollen competition or enhanced genetic sampling. In the present study, both seed quantity and quality increased with larger pollen loads, suggesting that declines in pollinator abundance may decrease plant fitness through lowered seed quality before an effect on seed set is detected.

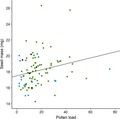

## 1 INTRODUCTION

As a consequence of the global decline in pollinators, many flowering plants are experiencing reduced pollination reliability and thus smaller amounts of pollen arriving onto stigmas (Eckert et al., 2009; Potts et al., 2010). Reduced stigmatic pollen loads (i.e., number of pollen grains arriving onto the stigmas) can negatively affect plant fitness by reducing both seed quantity and seed quality (Ashman et al., 2004; Burd, 1994; Labouche, Richards, & Pannell, 2017; Moeller, Geber, Eckhart, & Tiffin, 2012; Niesenbaum, 1999; Snow, 1982; Winsor, Davis, & Stephenson, 1987). Because a substantial pollen load is required to fertilize all ovules within a flower (Mitchell, 1997a; Snow, 1982), small pollen loads are often associated with low seed set, which is referred to as pollen limitation (Ashman et al., 2004). Under pollen limitation, each additional pollen grain may contribute to producing an additional seed, without necessarily affecting average seed quality (Burd, 1994; Mitchell, 1997a). Thus up to a point, increasing pollen loads will increase maternal fitness by increasing the number of seeds produced (Ashman et al., 2004; Mitchell, 1997b; Moeller et al., 2012; Snow, 1982; Winsor et al., 1987). The shape of the relationship between pollen load and seed set differs among species, populations, and even among individuals within a population (Mitchell, 1997b; Waser & Price, 1991). The shape of this relationship is of great importance, because it determines the minimum pollen load necessary to produce full seed set.

Pollen loads exceeding the pollen‐limitation threshold will lead to competition among pollen grains for ovule fertilization, which may improve offspring quality and maternal fitness. Competition among pollen from single or multiple pollen donors can positively affect offspring performance (Armbruster & Rogers, 2004; Labouche et al., 2017; Lankinen & Armbruster, 2007; Mulcahy & Mulcahy, 1975, 1987; Paschke, Abs, & Schmid, 2002; Richardson & Stephenson, 1992; Waser & Price, 1991; Winsor et al., 1987). Under pollen competition, high‐quality pollen grains are expected to outcompete low‐quality pollen grains in the race for ovule fertilization, hence decreasing stochasticity in the fertilization process and increasing average offspring quality. The outcome of pollen competition will depend on two processes. First, competition intensity will increase with increasing pollen‐load size, decreasing positional variance of the pollen grains on the stigmas, or decreasing variation in the timing of pollen arrival (Armbruster, Martin, Kidd, Stafford, & Rogers, 1995; Mitchell, 1997a; Mulcahy & Mulcahy, 1987; Niesenbaum, 1999; Winsor et al., 1987). Second, larger pollen loads may increase variation in the genetic quality of pollen grains. This may increase the probability for high‐quality pollen grains to be among the competing pollen grains (i.e., genetic sampling effect), and simultaneously decrease chances for low‐quality pollen grains to achieve fertilization (Mazer, Moghaddasi, Bello, & Hove, 2016; Paschke et al., 2002; Richardson & Stephenson, 1991; Waser & Price, 1991). Thus, variation in pollen‐load size affects both the opportunity for pollen competition and its intensity, because a larger pollen load provides more genetic variation in the pollen sample, but a less random sample with regard to genetic quality in the pollen reaching the ovules (Bernasconi, Paschke, & Schmid, 2003; Labouche et al., 2017; Mazer et al., 2016; Richardson & Stephenson, 1991; Waser & Price, 1991; Winsor et al., 1987).

Tests of the effects of pollen load on seed size are complicated by the seed‐number seed‐size trade‐off, where the seed size may increase when seed set is lower than the maximum number of seeds per flower (Labouche et al., 2017; Mulcahy & Mulcahy, 1987). This trade‐off may obscure the effects of pollen‐competition intensity or genetic sampling on seed‐quality traits like seed size (see discussion in Charlesworth, 1988).

Previous studies of pollen competition and its effect on seed quality in the mixed‐mating vine *Dalechampia scandens* (Euphorbiaceae) have yielded somewhat conflicting results. While Armbruster and Rogers (2004) observed an increase in seed mass with increasing intensity of competition among self‐pollen, Opedal, Armbruster, and Pélabon (2015) found no effect of increased competition intensity among either self‐ or cross‐pollen on offspring quantity or quality. The latter results were confirmed by Pélabon et al. (2016) who found no effect of variation in the dispersion of pollen on the stigmatic surface on seed mass and seedling performance. However, in contrast to Armbruster and Rogers (2004), Opedal et al. (2015) and Pélabon et al. (2016) did not control for the distance the pollen tubes grew.

In this study, we tested the consequences of variation in stigmatic cross‐pollen loads from a single pollen donor on seed set and seed mass. In contrast to the previous studies, we assessed not only the effects of the intensity of pollen competition, but also the effects of genetic sampling within pollen donors. We focused on competition among cross‐pollen grains, because pollinator declines may reduce the stigmatic cross‐pollen load. We used relatively small pollen loads, within the range of cross‐pollen loads usually observed in natural populations (Opedal et al., 2016). We first established the shape of the relationship between pollen load and seed set in a greenhouse population of *D. scandens* and compared it to the relationship observed in natural populations (Opedal et al., 2016; Pérez‐Barrales, Bolstad, Pélabon, Hansen, & Armbruster, 2013). Then, we tested the effect of increasing pollen loads on seed mass, while accounting for a possible trade‐off between seed mass and seed number.

## 2 MATERIALS AND METHODS

### 2.1 Study population

**Figure 1:**
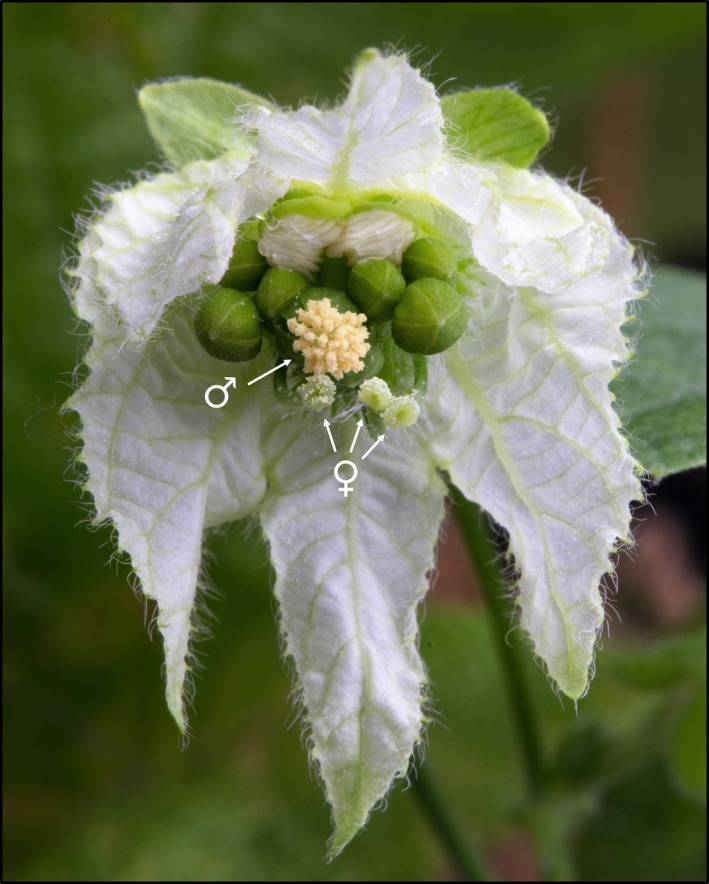
Blossom of *Dalechampia scandens* on the first day of the bisexual phase presenting one open male flower. Pollen grains are visible on the stigmas. Arrows indicate one open male flower and the stigmas of the three female flowers within the blossom. Photo: Per Harald Olsen

*Dalechampia scandens* L. (s.l.) (Euphorbiaceae) is a perennial monoecious vine occurring in the Neotropics (Armbruster, 1985). Blossoms (pseudanthia) comprise three female flowers and up to ten male flowers located in close proximity, rendering the blossoms functionally hermaphroditic (Figure 1). Female flowers contain three ovules each, and one blossom can bear a maximum of nine seeds. *Dalechampia scandens* is functionally protogynous, with a female phase that lasts for 2–4 days in our study population (mean = 3.00 days; *SD* = 0.65 days; Hildesheim, Opedal, Armbruster & Pélabon, 2019). With the opening of the first male flower, the blossom transitions into a bisexual phase lasting 2–4 days (mean = 3.65; *SD* = 0.67), during which the male flowers open in succession. The stigmas are receptive to pollination throughout the entire flowering period, and autonomous self‐pollination may occur during the bisexual phase (Opedal et al., 2015). In natural populations, stigmatic pollen loads during the female phase range from zero to more than 100 pollen grains per stigma (Opedal et al., 2016). Low herkogamy (short anther–stigma distance), which facilitates autonomous self‐pollination, is commonly seen in populations with low rates of cross‐pollen receipt (Opedal et al., 2015, 2016). The experimental plants originated from a population located near Tovar in the state of Mérida, Venezuela (8°21′N, 71°46′W). This population belongs to the small‐glanded taxon of the *D. scandens* species complex, characterized by blossoms with relatively small resin‐producing glands (Bolstad et al., 2014). It has relatively low herkogamy (mean = 2.63 mm; *SE* = 0.46 mm) and is hence assumed to have experienced low cross‐pollen loads in nature (Opedal et al., 2016). Fruits are dry capsules which dehisce explosively when fully mature, about 4 weeks after pollination (mean = 29.30 days; *SD* = 0.82 days).

### 2.2 Experimental design

Plants were grown under standardized conditions in a single room in the greenhouse at the Department of Biology, NTNU (Trondheim, Norway). Light conditions (13 hr light/11 hr dark) and temperature (26°C day/24°C night) were held constant during the study period (December 2016–March 2017). The study population was grown from seeds derived from random within‐population crosses in the greenhouse. For the experiment, we used 26 individuals with known pedigrees, belonging to the fourth greenhouse generation. The experimental individuals served repeatedly as mothers (*n* = 25), fathers (*n* = 19), or both. Designated female (pollen‐receiving) blossoms were emasculated by removing the male cymule (cluster of male flowers, including the resin gland) before the anthers dehisced to prevent autonomous self‐pollination. To exclude resource competition among the female flowers and minimize variation in seed size due to position within a blossom, we removed the stigmas of the two lateral female flowers and left only the unmanipulated middle flower for experimental pollination. Thus, we constrained blossom seed set to a maximum of three seeds. Flowers within a blossom are independent, and the removal of the stigma from the lateral flowers should not affect the remaining flower. We performed between one and eight crosses (mean = 4.28; *SD* = 1.88) per maternal plant and randomized the size of the pollen load among crosses on the same maternal individual. For pollination, we used pollen from a freshly dehisced male flower from a randomly chosen pollen donor. Note that effects of paternal identity on seed mass are very limited in *D. scandens* (Pélabon, Albertsen, Falahati‐Anbaran, Wright, & Armbruster, 2015; Pélabon et al., 2016).

We pollinated flowers by brushing plastic toothpicks over the anthers of the paternal individual and transferred the pollen grains with the toothpick onto the stigmas of the maternal individual. For each cross, we used a fresh toothpick to avoid contamination. We varied the pollen load by touching the stigma either once, or repeatedly, or by brushing the toothpick against the stigma. If pollen grains were clumped, we attempted to break up the clumps to ensure even contact of pollen grains with the stigmatic surface. This method produced a different number of pollen grains on the stigma for each cross. After pollination, one or two observers used hand lenses to count the number of pollen grains adhering to the stigma. In this way, we placed between 1 and 76 pollen grains on the stigma, which represents a range of pollen load similar to the range observed in natural populations (Opedal et al., 2016). The stigmas of *D. scandens* extend down the lateral surface of the style, and pollen grains placed on the lateral stigmatic surface grow pollen tubes that are equally efficient at fertilizing seeds as pollen placed on the stigma tip (Armbruster et al., 1995). Therefore, the intensity of pollen competition may vary due to variation in the distance a pollen tube must grow to achieve ovule fertilization (Armbruster & Rogers, 2004; Opedal et al., 2015; Pélabon et al., 2016). To control for such variation, we placed pollen primarily on the stigma tip. However, especially at larger pollen loads, we could not avoid pollen grains landing on the extended stigmatic surface. Therefore, we counted pollen both on the stigma tip and on the extended stigmatic surface to determine the total number of pollen grains. Pollen counts were repeated three times, and we treated the average of the three repeated counts as the measure of pollen load.

Previous studies of different taxa of the *D. scandens* complex showed that blossom size may affect seed mass (Opedal et al., 2015; Pélabon et al., 2015, 2016). To account for this source of variation, we measured the diameter of the peduncle of the pollinated blossoms with digital callipers (0.01 mm precision) and used this measure as a proxy for blossom size. After hand‐pollination, we bagged the blossoms to collect the seeds when the matured seed capsules dehisced. For each blossom, we counted the seeds produced and weighed them individually to the nearest 0.1 mg on a precision balance. We used seed mass as our measure of offspring quality, because of the positive relationship between seed mass and offspring fitness observed in our study species (Armbruster & Rogers, 2004; Pélabon, Carlson, Hansen, & Armbruster, 2005), as well as other species (Labouche et al., 2017; Winsor et al., 1987). We weighed all seeds shortly after capsule dehiscence to minimize variation in seed mass due to water loss. For analysis, we considered the average seed mass per flower, which in this experiment represents a whole blossom. Underdeveloped seeds (average mass of 3.75 mg, *n* = 2) occurred in two seed sets. We included them for the analyses of seed number per seed set, but removed them from analyses of seed mass.

### 2.3 Statistical analyses

All statistical analyses were performed with R version 3.3.1 (R Core Team, 2016). We modeled the effect of pollen load on seed set using the asymptotic relationship: $\text{Seed set}=3\frac{\alpha \left(\text{pollen load}\right)}{1+\alpha \left(\text{pollen load}\right)}$, where pollen load is the average of the three repeated pollen counts, and α is a shape parameter describing how rapidly seed set increases with pollen load. Multiplication by three relates to the maximum seed set one flower can produce. Following Pérez‐Barrales et al. (2013), we estimated α as the natural exponent of the intercept of a generalized linear mixed‐effect model, with the probability of a seed being fertilized predicted by pollen load, and the slope set to one by treating pollen load as an offset variable. Pollen load was natural log‐transformed before the analysis, and maternal plant was treated as a random factor. We fitted the model with a logit link and binomial error distribution. The repeatability of the pollen counts estimated with the “rptR” R‐package (Stoffel, Nakagawa, & Schielzeth, 2017) was high (*r* = 0.92; 95% CI = 0.89; 0.95). We calculated the seed:pollen ratio as the average of the within‐flower ratios.

We tested the effects of pollen load, peduncle diameter, and seed set (number of seeds per fruit) on seed mass (average seed mass per flower) by comparing models with the Akaike information criterion corrected for small sample size (AICc). We fitted mixed‐effect models with seed mass as the response variable, pollen load, peduncle diameter, and seed set as explanatory variables, and maternal plant as a random factor. Including paternal identity as a random factor did not improve the fit of the model (not shown), and paternal identity was, therefore, not included. We compared models fitted with maximum likelihood (ML) with the “lme4” R‐package (Bates, Maechler, Bolker, & Walker, 2015) and obtained parameter estimates for the highest ranked models fitted with restricted maximum likelihood (REML).

## 3 RESULTS

**Figure 2:**
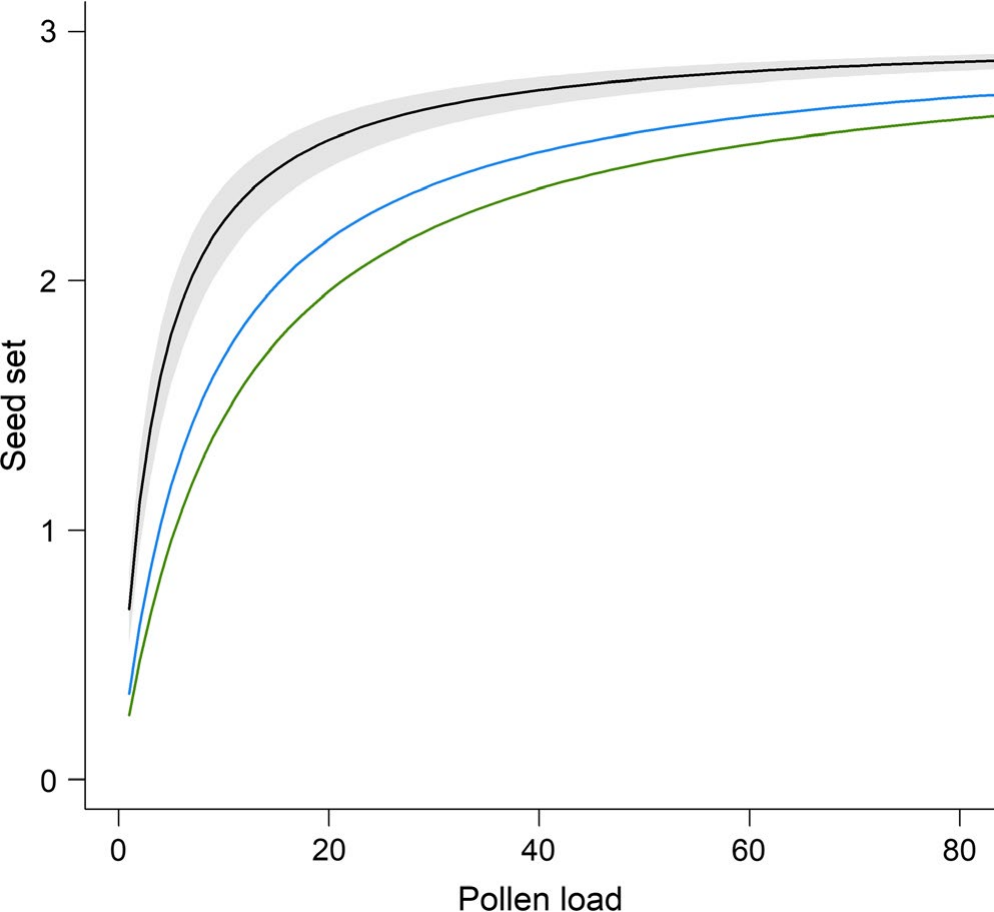
Seed set in response to variation in pollen load on stigmas of *Dalechampia scandens* flowers in the greenhouse, representing the asymptotic function: $\text{Seed set}=3\frac{0.29\left(\text{pollen load}\right)}{1+0.29\left(\text{pollen load}\right)}$ (black line). The shaded area represents the 95% confidence interval of the function. The green line is the relationship established by Pérez‐Barrales et al. (2013; *α* = 0.094) in a natural Mexican population, and the blue line is the mean relationship established by Opedal et al. (2016; *α* = 0.130) for four natural populations of *D. scandens* in Costa Rica

Across all crosses (*n* = 107 crosses on 25 maternal plants), the pollen load ranged from 1 to 76 pollen grains, with an average of 16.1 (median = 13.0; *SD* = 13.0). About 17% of the flowers failed to set seed, despite an average pollen load of 9.2 pollen grains on these flowers (*SD* = 10.9; Table 1). Unsuccessful crosses were distributed randomly among maternal plants. The relationship between pollen load and seed set followed an asymptotic curve, with the shape parameter *α* = 0.29 (95% CI = 0.23; 0.39) describing the odds of producing a seed per pollen grain (Figure 2). This value of α translates two pollen grains into 1.11 seeds (95% CI = 0.93; 1.31), seven pollen grains into 2.02 seeds (1.84; 2.19), and 22 pollen grains into 2.60 seeds (2.50; 2.68), resulting in one, two, or three seeds. This conversion rate is higher than those obtained in natural *D. scandens* populations (Opedal et al., 2016; Pérez‐Barrales et al., 2013; Figure 2). Flowers producing full seed set of three seeds received on average 18.6 (*SD* = 11.5) pollen grains. The average seed:pollen ratio across all seed sets was 0.21 and ranged from 0.11 to 0.40 seeds per pollen grain (Table 1).

**Table 1:** Summary statistics for pollen load and seed mass for each seed‐set number in *Dalechampia scandens*

Seed set	Observations	Pollen load				Seed mass (mg)				Seed:pollen ratio	
		Mean	*SD*	Min.	Max.	Mean	*SD*	Min.	Max.	Mean	*SE*
Overall	107	16.14	13.03	1	75.67	18.16	2.04	14.27	26.33	0.21	0.021
0	18	9.24	10.94	1	45.67	NA	NA	NA	NA	0	0
1	6	10.78	3.72	4.67	16	18.02	1.76	16.00	20.20	0.11	0.022
2	16	15.65	19.47	2	75.67	17.37	1.65	15.00	20.25	0.40	0.090
3	67	18.60	11.52	3.33	56.67	18.36	2.12	14.27	26.33	0.23	0.020

**Figure 3:**
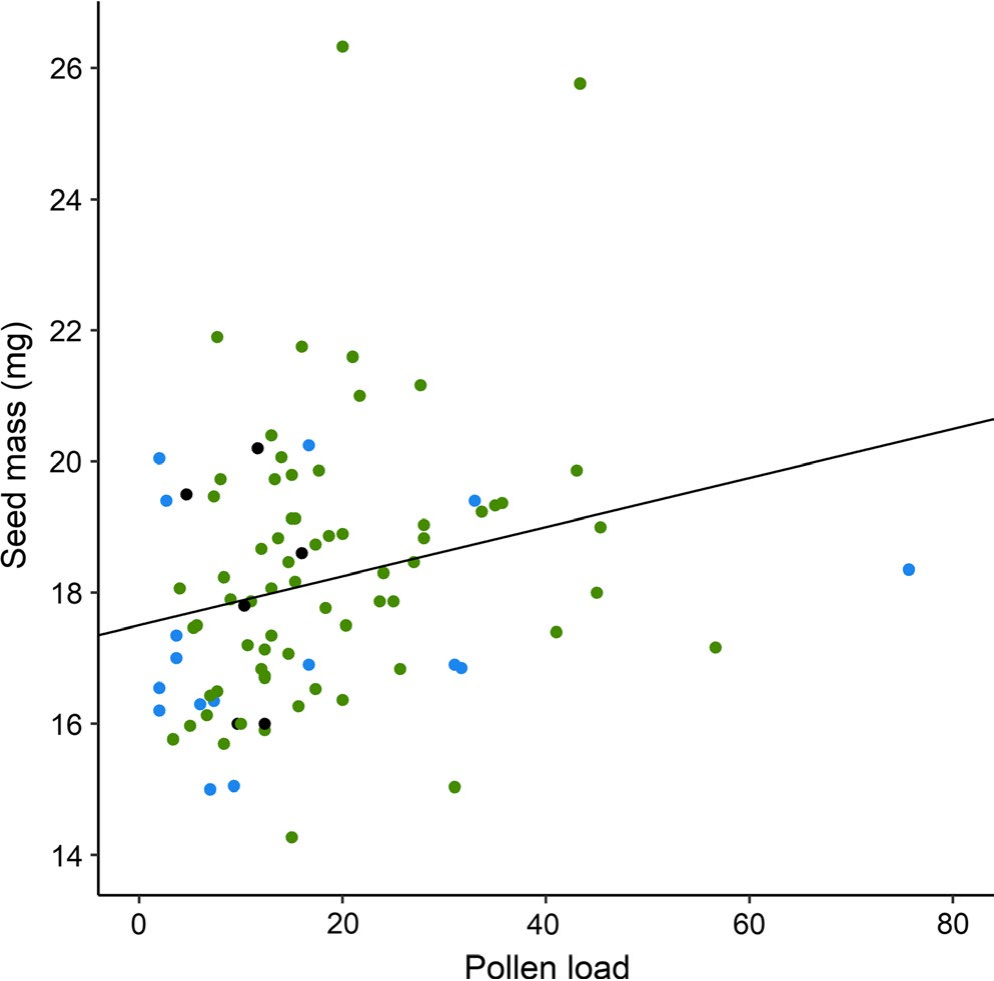
Relationship between pollen load and seed mass in *Dalechampia scandens*, *r* ^2^ = 0.06. Points represent mean seed mass (mg) per flower. Black: seed set = 1; blue: seed set = 2; and green: seed set = 3

The mean seed mass (*n* = 239 seeds from 107 flowers) was 18.16 mg (*SD* = 2.04) and ranged from 14.27 mg to 26.33 mg, excluding underdeveloped seeds (Table 1). We did not detect a trade‐off between seed mass and seed number (Table 2). Variation in seed mass did not increase with seed number (a model allowing for different residual variances across seed‐set categories did not fit the data any better; not shown) or pollen load (inspection of this relationship did not reveal a statistically significant effect of pollen load on variance in seed mass; not shown). Pollen load had a relatively small, but statistically significant, positive effect on seed mass (Tables 2 and 3). Seed mass increased by 2.4% per standard deviation increase in pollen load. Overall, 6% of the variation in seed mass could be attributed to variation in pollen load (Figure 3). There were weak positive effects of peduncle diameter and seed set on seed mass, but neither of these were supported statistically (Tables 2 and 3).

**Table 2:** Parameter estimates from the highest ranked models with seed mass as response variable, and pollen load, peduncle diameter, and seed set as predictor variables

Model	Parameter	Estimate	*SE*	*T*‐value	95% CI
Seed mass ~ Pollen load	Intercept (mg)	17.57	0.40	44.18	16.79, 18.35
	Pollen load (mg/pollen grain)	0.03	0.01	2.46	0.01, 0.06
Seed mass ~ Peduncle + Pollen load	Intercept (mg)	16.10	1.45	11.08	13.17, 18.96
	Peduncle (mg/mm)	1.74	1.67	1.04	−1.54, 5.14
	Pollen load (mg/pollen grain)	0.03	0.01	2.31	0, 0.06
Seed mass ~ Seed set + Pollen load	Intercept (mg)	16.89	0.83	20.42	15.27, 18.50
	Seed set (mg/seed)	0.26	0.28	0.93	−0.29, 0.81
	Pollen load (mg/pollen grain)	0.03	0.01	2.28	0, 0.06

Note

Parameters were obtained from models fitted with restricted maximum likelihood (REML). Maternal identity was included as a random factor in all models.

**Table 3:** Model comparison for the effect of pollen load, peduncle diameter, and seed set on seed mass in *Dalechampia scandens*

Model	Parameters (K)	AICc	ΔAICc	AICc weight
Seed mass ~ Pollen load	4	357.74	0	0.30
Seed mass ~ Peduncle + Pollen load	5	358.88	1.14	0.17
Seed mass ~ Seed set + Pollen load	5	359.10	1.36	0.15
Seed mass ~ Peduncle + Seed set + Pollen load	6	359.77	2.03	0.11
Seed mass ~ Peduncle + Seed set × Pollen load	7	359.93	2.19	0.10
Seed mass ~ 1	3	361.53	3.79	0.05
Seed mass ~ Peduncle + Seed set	5	361.80	4.06	0.04
Seed mass ~ Peduncle	4	361.99	4.25	0.04
Seed mass ~ Seed set	4	362.07	4.33	0.03

Note

Models are ranked by increasing AICc values obtained from mixed‐effect models fitted with maximum likelihood (ML). Maternal identity was included as a random factor in all models.

## 4 DISCUSSION

Understanding how variation in stigmatic pollen load affects seed production is important in the light of current pollinator declines and their possible effects on plant fitness. We detected a relatively low pollen‐load threshold associated with a full seed set in a greenhouse population of *Dalechampia scandens*. The average seed:pollen ratio was 0.21 (*SE* ± 0.021), comparable to the mean seed:pollen ratios of 0.27–0.38 observed in several studies reporting effects of low pollen‐load sizes on offspring fitness (Mitchell, 1997a). The steep increase in seed number at small pollen loads may result from the plants' tendency to prioritize the fertilization of all ovules to maximize seed set, regardless of pollen quality. Similar asymptotic relationships between pollen load and seed set have been established previously (Ashman et al., 2004; Mitchell, 1997b; Waser & Price, 1991). The shape parameter observed in this experiment (*α* = 0.29) was larger than those observed in natural populations of *D. scandens* (0.094 and 0.130 in Pérez‐Barrales et al. (2013) and Opedal et al. (2016), respectively). This suggests that the translation of pollen load into seed set is less efficient and pollen limitation more severe in nature, than predicted by our greenhouse experiment. This may result from various environmental differences, including maternal resource limitation, the effect of older, less vigorous pollen arriving onto stigmas in the wild, as well as genetic effects due to self‐pollination. Furthermore, in the present study, we tested pollen competition within a single paternal individual from a single pollination event, whereas pollination by multiple pollen donors, with variation in the timing of pollen deposition, may be more likely in nature. In this case, poor‐quality pollen grains could exclude late arriving high‐quality pollen grains from ovule fertilization, thus diminishing the rate of increase in seed set with increasing pollen numbers in natural populations, compared with our experimental population (Harder, Aizen, & Richards, 2016). Finally, we constrained blossom seed set to a maximum of three seeds, possibly increasing the chances of these seeds to mature compared to the possible nine seeds produced in natural populations. Because most natural populations of *D. scandens* receive fewer than ten cross‐pollen grains per stigma, on average (Opedal et al., 2016), further reduction in pollination reliability may negatively affect the reproductive capacity of populations through lowered production of outcrossed seeds. Mixed‐mating species may be able to compensate for reductions in cross‐pollen receipt via autonomous self‐pollination, thus mitigating the fitness loss due to outcross pollen limitation (Eckert et al., 2009; Kalisz, Vogler, & Hanley, 2004). Moderate rates of cross‐pollination combined with low outcrossing rates in most natural populations of *D. scandens* suggest that a substantial proportion of seeds result from autonomous selfing (Opedal et al., 2016).

Although three pollen grains per stigma should be sufficient to achieve a full seed set (three seeds) in *D. scandens*, we observed that 19 pollen grains were required on average. This might be due to a minimum pollen load necessary to initiate pollen‐tube germination (Brewbaker & Kwack, 1963), and positive density‐dependent effects facilitating pollen germination at moderate densities (Harder et al., 2016). Furthermore, pollen mortality due to physiological causes or poor genetic quality may eliminate pollen grains before the onset of pollen‐tube competition (Harder et al., 2016). It also suggests that pollen competition may occur even before full seed set is reached. We failed to detect a trade‐off between seed number and seed mass within fruits (Table 2), or an interaction between pollen load and seed set in the model testing effects on seed mass (Table 3). Therefore, the simultaneous increases in seed number and seed mass with pollen load seem to occur independently of one another. In perennial species, such as *D. scandens*, where each plant produces a large number of blossoms during its lifetime and the number of flowers per individual may vary greatly, a trade‐off between seed mass and seed number may be more likely expressed among rather than within blossoms (Burd, 1994; Labouche et al., 2017; Pélabon et al., 2015).

The observed positive relationship between pollen load and seed mass may be caused by pollen competition, which is the nonrandom fertilization of ovules by pollen with faster‐growing pollen tubes (Labouche et al., 2017; Winsor et al., 1987). The effect of pollen competition on fitness depends on the intensity of pollen competition and the variation in genetic quality among pollen grains. The former is affected by, among other things, the mean and variance of the distance travelled by competing pollen tubes and the pollen grains per ovule ratio. The latter is strongly linked to the size of pollen loads, because larger pollen loads are more likely to include high‐quality pollen (genetic sampling effect), regardless of the number of pollen donors (Bernasconi et al., 2003; Mazer et al., 2016; Winsor et al., 1987). Studies assessing the effect of pollen‐competition intensity on seed quality without variation in genetic sampling (by manipulating positional variance of pollen loads of roughly equal size) in *D. scandens* have yielded conflicting results. Armbruster and Rogers (2004) reported higher seed mass and better performance of seedlings from seeds produced under stronger self‐pollen competition, while later studies on different populations did not detect such an effect with self‐ or cross‐pollen (Opedal et al., 2015; Pélabon et al., 2016). By holding pollen‐load size and genetic diversity constant, these studies prevented genetic sampling from influencing seed quality. In the present study, by varying pollen loads and thereby genetic sampling and competition intensity within a single pollen donor, we found a small but detectable positive effect on seed mass, even at relatively low pollen loads. Compared with previous studies (Mitchell, 1997a; Pélabon et al., 2016; Waser & Price, 1991), our results suggest that genetic sampling effects on pollen competition may be most readily detected at low to moderate pollen loads, because at high pollen loads, high‐quality pollen grains may be sufficiently abundant to fertilize all available ovules. This may explain why the study by Pélabon et al. (2016), which applied pollen loads two to three times larger than the largest pollen load used in the present study, failed to find any effect of pollen competition on seed mass. Taken together with the studies of Opedal et al. (2015) and Pélabon et al. (2016), the current study suggests that, in *D. scandens* and most likely in many other species, seed mass is most strongly affected by differences in genetic sampling and competition intensity at relatively low pollen loads. Competition among pollen from multiple donors likely magnifies this effect by increasing genetic variation and hence differences in quality among the pollen grains.

Several studies have failed to detect direct effects of pollen competition on offspring quality, particularly when measured as seed mass (Mitchell, 1997a; b; Niesenbaum, 1999; Richardson & Stephenson, 1991; Snow, 1990). For example, Niesenbaum (1999) found a positive effect of pollen‐load size on seed set, but not on seed mass in *Mirabilis jalapa*. Richardson and Stephenson (1991) did not find a statistically significant effect of pollen‐load size on seed set or seed mass in *Campanula americana*. However, a positive effect of pollen competition on seed mass could have been masked by a negative correlation between seed set and seed mass. In some studies, pollen‐competition effects on offspring performance were detected after accounting for the seed‐number seed‐mass trade‐off (Labouche et al., 2017; Winsor et al., 1987). In other studies, pollen competition had no detectable effect on seed mass, but influenced later offspring performance (Mulcahy & Mulcahy, 1975; Richardson & Stephenson, 1992). Thus, if genetic sampling or competition intensity also affect positively later life stages, our experiment may underestimate the effects of larger pollen load on offspring fitness.

In conclusion, our study confirms that increasing pollen loads, even from a single donor, may positively affect the fitness of the maternal plant via an increase in seed quality, both below and above levels achieving full seed set. These results are particularly important in the context of current pollinator declines observed worldwide, because they suggest that declines in pollination reliability may negatively affect plant fitness before any effect on seed set is detected. Our experiment also highlights the necessity of studying these effects in natural populations, because greenhouse studies may lead to underestimation of the severity of cross‐pollen limitation in nature.

## CONFLICT OF INTEREST

None declared.

## AUTHOR CONTRIBUTIONS

LSH, ØHO, and CP conceived and designed the study. LSH collected the data. LSH, ØHO, and CP analyzed the data. LSH led the writing, and all authors contributed to revisions.

## Data Availability

Data supporting the results is stored in the Dryad Data Repository (https://doi.org/10.5061/dryad.bg79cnp72).

## References

[ece35858-bib-0001] Armbruster, W. S. (1985). Patterns of character divergence and the evolution of reproductive ecotypes of *Dalechampia* *scandens* (Euphorbiaceae). Evolution, 39, 733–752. 28561369 10.1111/j.1558-5646.1985.tb00416.x

[ece35858-bib-0002] Armbruster, W. S. , Martin, P. , Kidd, J. , Stafford, R. , & Rogers, D. G. (1995). Reproductive significance of indirect pollen‐tube growth in *Dalechampia* (Euphorbiaceae). American Journal of Botany, 82, 51–56.

[ece35858-bib-0003] Armbruster, W. S. , & Rogers, D. G. (2004). Does pollen competition reduce the cost of inbreeding? American Journal of Botany, 91, 1939–1943. 10.3732/ajb.91.11.1939 21652341

[ece35858-bib-0004] Ashman, T.‐L. , Knight, T. M. , Steets, J. A. , Amarasekare, P. , Burd, M. , Campbell, D. R. , … Wilson, W. G. (2004). Pollen limitation of plant reproduction: Ecological and evolutionary causes and consequences. Ecology, 85, 2408–2421. 10.1890/03-8024

[ece35858-bib-0005] Bates, D. , Maechler, M. , Bolker, B. , & Walker, S. (2015). Fitting linear mixed‐effects models using lme4. Journal of Statistical Software, 67, 1–48.

[ece35858-bib-0006] Bernasconi, G. , Paschke, M. , & Schmid, B. (2003). Diversity effects in reproductive biology. Oikos, 102, 217–220. 10.1034/j.1600-0706.2003.12598.x

[ece35858-bib-0007] Bolstad, G. H. , Hansen, T. F. , Pélabon, C. , Falahati‐Anbaran, M. , Pérez‐Barrales, R. , & Armbruster, W. S. (2014). Genetic constraints predict evolutionary divergence in *Dalechampia* blossoms. Philosophical Transactions of the Royal Society of London. Series B, Biological Sciences, 369, 20130255. 25002700 10.1098/rstb.2013.0255 PMC4084540

[ece35858-bib-0008] Brewbaker, J. L. , & Kwack, B. H. (1963). The essential role of calcium ion in pollen germination and pollen tube growth. American Journal of Botany, 50, 859–865. 10.1002/j.1537-2197.1963.tb06564.x

[ece35858-bib-0009] Burd, M. (1994). Bateman's principle and plant reproduction: The role of pollen limitation in fruit and seed set. The Botanical Review, 60, 83–139. 10.1007/BF02856594

[ece35858-bib-0010] Charlesworth, D. (1988). Evidence for pollen competition in plants and its relationship to progeny fitness: A comment. The American Naturalist, 132, 298–302. 10.1086/284852

[ece35858-bib-0011] Eckert, C. G. , Kalisz, S. , Geber, M. A. , Sargent, R. , Elle, E. , Cheptou, P.‐O. , … Winn, A. A. (2009). Plant mating systems in a changing world. Trends in Ecology and Evolution, 25, 35–43. 10.1016/j.tree.2009.06.013 19683360

[ece35858-bib-0012] Harder, L. D. , Aizen, M. A. , & Richards, S. A. (2016). The population ecology of male gametophytes: The link between pollination and seed production. Ecology Letters, 19, 497–509. 10.1111/ele.12596 26970246

[ece35858-bib-0013] Hildesheim, L. S. , Opedal, Ø. H. , Armbruster, W. S. , & Pélabon, C. (2019). Fitness costs of delayed pollination in a mixed‐mating plant. Annals of Botany, 10.1093/aob/mcz141. [Epub ahead of print]. PMC6868360 31504153

[ece35858-bib-0014] Kalisz, S. , Vogler, D. W. , & Hanley, K. M. (2004). Context‐dependent autonomous self‐fertilization yields reproductive assurance and mixed mating. Nature, 430, 884–887. 10.1038/nature02776 15318220

[ece35858-bib-0015] Labouche, A.‐M. , Richards, S. A. , & Pannell, J. R. (2017). Effects of pollination intensity on offspring number and quality in a wind‐pollinated herb. Journal of Ecology, 105, 197–208. 10.1111/1365-2745.12659

[ece35858-bib-0016] Lankinen, Å. , & Armbruster, W. S. (2007). Pollen competition reduces inbreeding depression in *Collinsia* *heterophylla* (Plantaginaceae). Journal of Evolutionary Biology, 20, 737–749. 10.1111/j.1420-9101.2006.01233.x 17305839

[ece35858-bib-0017] Mazer, S. J. , Moghaddasi, A. , Bello, A. K. , & Hove, A. A. (2016). Winning in style: Longer styles receive more pollen, but style length does not affect pollen attrition in wild *Clarkia* populations. American Journal of Botany, 103, 408–422. 26933011 10.3732/ajb.1500192

[ece35858-bib-0018] Mitchell, R. J. (1997a). Effects of pollen quantity on progeny vigor: Evidence from the desert mustard *Lesquerella* *fendleri* . Evolution, 51, 1679–1684. 28568636 10.1111/j.1558-5646.1997.tb01490.x

[ece35858-bib-0019] Mitchell, R. J. (1997b). Effects of pollination intensity on *Lesquerella* *fendleri* seed set: Variation among plants. Oecologia, 109, 382–388. 10.1007/s004420050097 28307535

[ece35858-bib-0020] Moeller, D. A. , Geber, M. A. , Eckhart, V. M. , & Tiffin, P. (2012). Reduced pollinator service and elevated pollen limitation at the geographic range limit of an annual plant. Ecology, 93, 1036–1048. 10.1890/11-1462.1 22764490

[ece35858-bib-0021] Mulcahy, D. L. , & Mulcahy, G. B. (1975). The influence of gametophytic competition on sporophytic quality in *Dianthus * *chinensis* . Theoretical and Applied Genetics, 46, 277–280. 10.1007/BF00281149 24420121

[ece35858-bib-0022] Mulcahy, D. L. , & Mulcahy, G. B. (1987). The effects of pollen competition. American Scientist, 75, 44–50.

[ece35858-bib-0023] Niesenbaum, R. A. (1999). The effects of pollen load size and donor diversity on pollen performance, selective abortion, and progeny vigor in *Mirabilis * *jalapa* (Nyctaginaceae). American Journal of Botany, 86, 261–268. 21680363

[ece35858-bib-0024] Opedal, Ø. H. , Albertsen, E. , Armbruster, W. S. , Pérez‐Barrales, R. , Falahati‐Anbaran, M. , & Pélabon, C. (2016). Evolutionary consequences of ecological factors: Pollinator reliability predicts mating‐system traits of a perennial plant. Ecology Letters, 19, 1486–1495. 10.1111/ele.12701 27882704

[ece35858-bib-0025] Opedal, Ø. H. , Armbruster, W. S. , & Pélabon, C. (2015). Inbreeding effects in a mixed‐mating vine: Effects of mating history, pollen competition and stress on the cost of inbreeding. Annals of Botany Plants, 7, 1–13. 10.1093/aobpla/plv133 PMC4683981 26578744

[ece35858-bib-0026] Paschke, M. , Abs, C. , & Schmid, B. (2002). Effects of population size and pollen diversity on reproductive success and offspring size in the narrow endemic *Cochlearia* *bavarica* (Brassicaceae). American Journal of Botany, 89, 1250–1259. 10.3732/ajb.89.8.1250 21665726

[ece35858-bib-0027] Pélabon, C. , Albertsen, E. , Falahati‐Anbaran, M. , Wright, J. , & Armbruster, W. S. (2015). Does multiple paternity affect seed mass in angiosperms? An experimental test in *Dalechampia* *scandens* . Journal of Evolutionary Biology, 28, 1719–1733. 26174371 10.1111/jeb.12692

[ece35858-bib-0028] Pélabon, C. , Carlson, M. L. , Hansen, T. F. , & Armbruster, W. S. (2005). Effects of crossing distance on offspring fitness and developmental stability in *Dalechampia* *scandens* (Euphorbiaceae). American Journal of Botany, 92, 842–851. 10.3732/ajb.92.5.842 21652465

[ece35858-bib-0029] Pélabon, C. , Hennet, L. , Bolstad, G. H. , Albertsen, E. , Opedal, Ø. H. , Ekrem, R. K. , & Armbruster, W. S. (2016). Does stronger pollen competition improve offspring fitness when pollen load does not vary? American Journal of Botany, 103, 522–531. 10.3732/ajb.1500126 26451034

[ece35858-bib-0030] Pérez‐Barrales, R. , Bolstad, G. H. , Pélabon, C. , Hansen, T. F. , & Armbruster, W. S. (2013). Pollinators and seed predators generate conflicting selection on *Dalechampia* blossoms. Oikos, 122, 1411–1428.

[ece35858-bib-0031] Potts, S. G. , Biesmeijer, J. C. , Kremen, C. , Neumann, P. , Schweiger, O. , & Kunin, W. E. (2010). Global pollinator declines: Trends, impacts and drivers. Trends in Ecology and Evolution, 25, 345–353. 20188434 10.1016/j.tree.2010.01.007

[ece35858-bib-0032] R Core Team . (2016). R: A language and environment for statistical computing. Vienna, Austria: R Foundation for Statistical Computing.

[ece35858-bib-0033] Richardson, T. E. , & Stephenson, A. G. (1991). Effects of parentage, prior fruit set and pollen load on fruit and seed production in *Campanula * *americana* L. Oecologia, 87, 80–85. 28313355 10.1007/BF00323783

[ece35858-bib-0034] Richardson, T. E. , & Stephenson, A. G. (1992). Effects of parentage and size of the pollen load on progeny performance in *Campanula * *americana* . Evolution, 46, 1731–1739. 28567761 10.1111/j.1558-5646.1992.tb01165.x

[ece35858-bib-0035] Snow, A. A. (1982). Pollination intensity and potential seed set in *Passiflora * *vitifolia* . Oecologia, 55, 231–237. 10.1007/BF00384492 28311238

[ece35858-bib-0036] Snow, A. A. (1990). Effects of pollen‐load size and number of donors on sporophyte fitness in wild radish (*Raphanus* *raphanistrum*). The American Naturalist, 136, 742–758. 10.1086/285129

[ece35858-bib-0037] Stoffel, M. A. , Nakagawa, S. , & Schielzeth, H. (2017). rptR: Repeatability estimation and variance decomposition by generalized linear mixed‐effects models. Methods in Ecology and Evolution, 8, 1639–1644. 10.1111/2041-210X.12797

[ece35858-bib-0038] Waser, N. M. , & Price, M. V. (1991). Outcrossing distance effects in *Delphinium * *nelsonii*: Pollen loads, pollen tube, and seed set. Ecology, 72, 171–179.

[ece35858-bib-0039] Winsor, J. A. , Davis, L. E. , & Stephenson, A. G. (1987). The relationship between pollen load and fruit maturation and the effect of pollen load on offspring vigor in *Cucurbita* * pepo* . The American Naturalist, 129, 643–656. 10.1086/284664

